# Optimizing knee osteoarthritis severity prediction on MRI images using deep stacking ensemble technique

**DOI:** 10.1038/s41598-024-78203-x

**Published:** 2024-11-05

**Authors:** Punita Panwar, Sandeep Chaurasia, Jayesh Gangrade, Ashwani Bilandi, Dayananda Pruthviraja

**Affiliations:** 1 Department of Computer Science & Engineering, School of Computer Science & Engineering, Manipal University Jaipur, Jaipur, Rajasthan India; 2 Department of Artificial Intelligence & Machine Learning, Computer Science & Engineering, Manipal University Jaipur, Jaipur, Rajasthan India; 3 Department of Orthopedics , MBBS, Mahatma Gandhi Medical College, Jaipur, Rajasthan India; 4 Department of Information Technology, Manipal Institute of Technology Bengaluru, Manipal Academy of Higher Education, Manipal, Karnataka India

**Keywords:** Knee osteoarthritis, Magnetic resonance imaging (MRI), Deep learning algorithms, Convolutional neural network, Deep Stack Ensemble, Bone, Ageing

## Abstract

Knee osteoarthritis (KOA) represents a well-documented degenerative arthropathy prevalent among the elderly population. KOA is a persistent condition, also referred to as progressive joint Disease, stemming from the continual deterioration of cartilage. Predominantly afflicting individuals aged 45 and above, this ailment is commonly labeled as a “wear and tear” joint disorder, targeting joints such as the knee, hand, hips, and spine. Osteoarthritis symptoms typically increase gradually, contributing to the deterioration of articular cartilage. Prominent indicators encompass pain, stiffness, tenderness, swelling, and the development of bone spurs. Diagnosis typically involves the utilization of Radiographic X-ray images, Magnetic Resonance Imaging (MRI), and Computed Tomography (CT) Scan by medical professionals and experts. However, this conventional approach is time-consuming, and also sometimes tedious for medical professionals. In order to address the limitation of time and expedite the diagnostic process, deep learning algorithms have been implemented in the medical field. In the present investigation, four pre-trained models, specifically CNN, AlexNet, ResNet34 and ResNet-50, were utilized to predict the severity of KOA. Further, a Deep stack ensemble technique was employed to achieve optimal performance resulting to the accuracy of 99.71%.

## Introduction

**Fig. 1:**
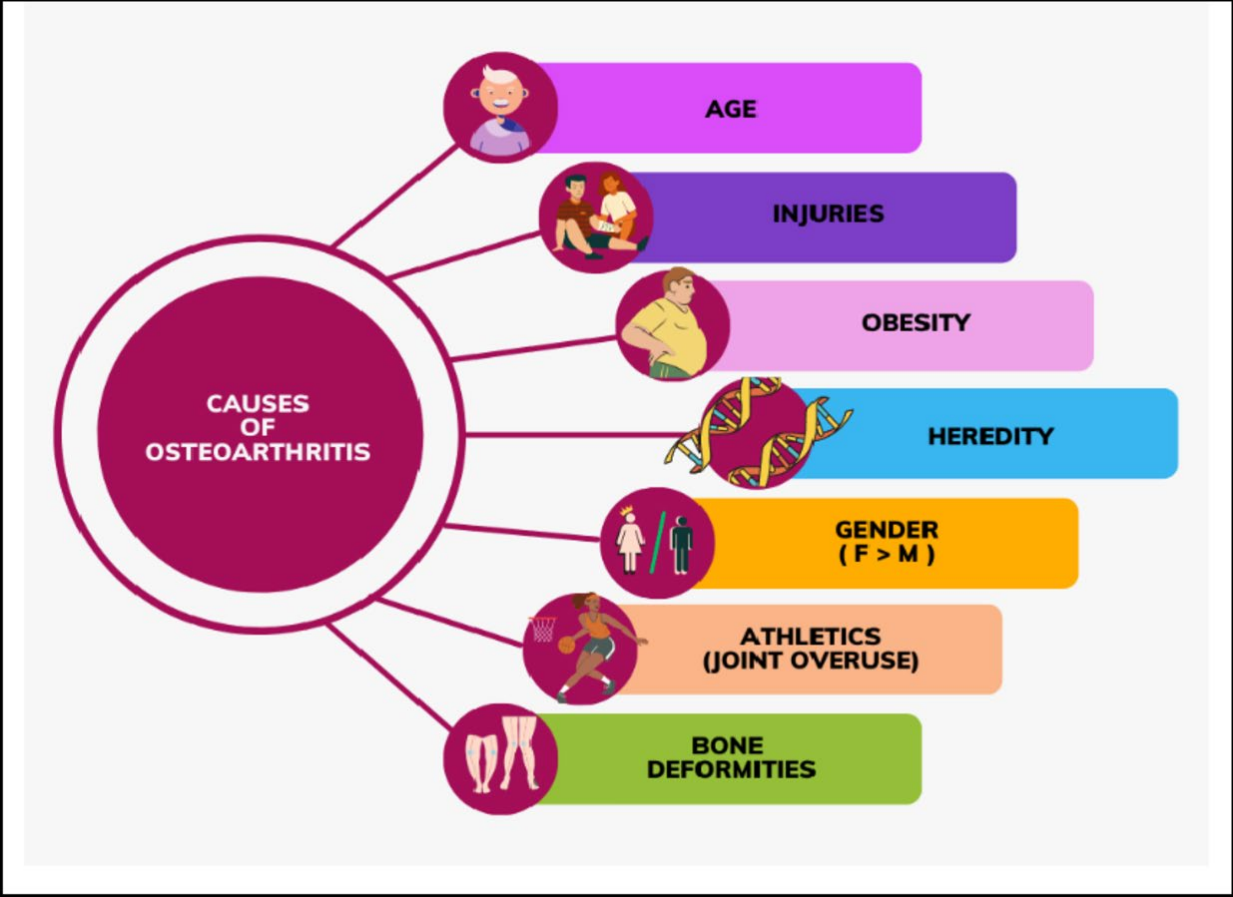
Causes of Knee Osteoarthritis.

Knee Osteoarthritis (KOA) is a progressive degenerative disorder resulting from mechanical strain on the knee joint, driven by an aging population and obesity epidemic. Symptomatic KOA occurs in approximately 240 cases per 100,000 people annually^1^. This disease gradually erodes the knee joint over 10 to 15 years, affecting all its divisions and leading to operational disability and decreased quality of life, primarily among older individuals of age 45 and above^2^. The rate of advancement and intensity of clinical manifestations can vary among individuals. Excessive weight on the knee, combined with factors like age, diabetes, inflammation, and misalignment, can severely impact knee function. However, these variations primarily manifest when there is a commencement of deterioration in the articular cartilage, accompanied by the formation of osteophytes near the joints. Gender, obesity, age, bone abnormalities, trauma, heredity, and lifestyle represent the most significant factors influencing KOA, as illustrated in Fig. 1^3^.

**Fig. 2:**
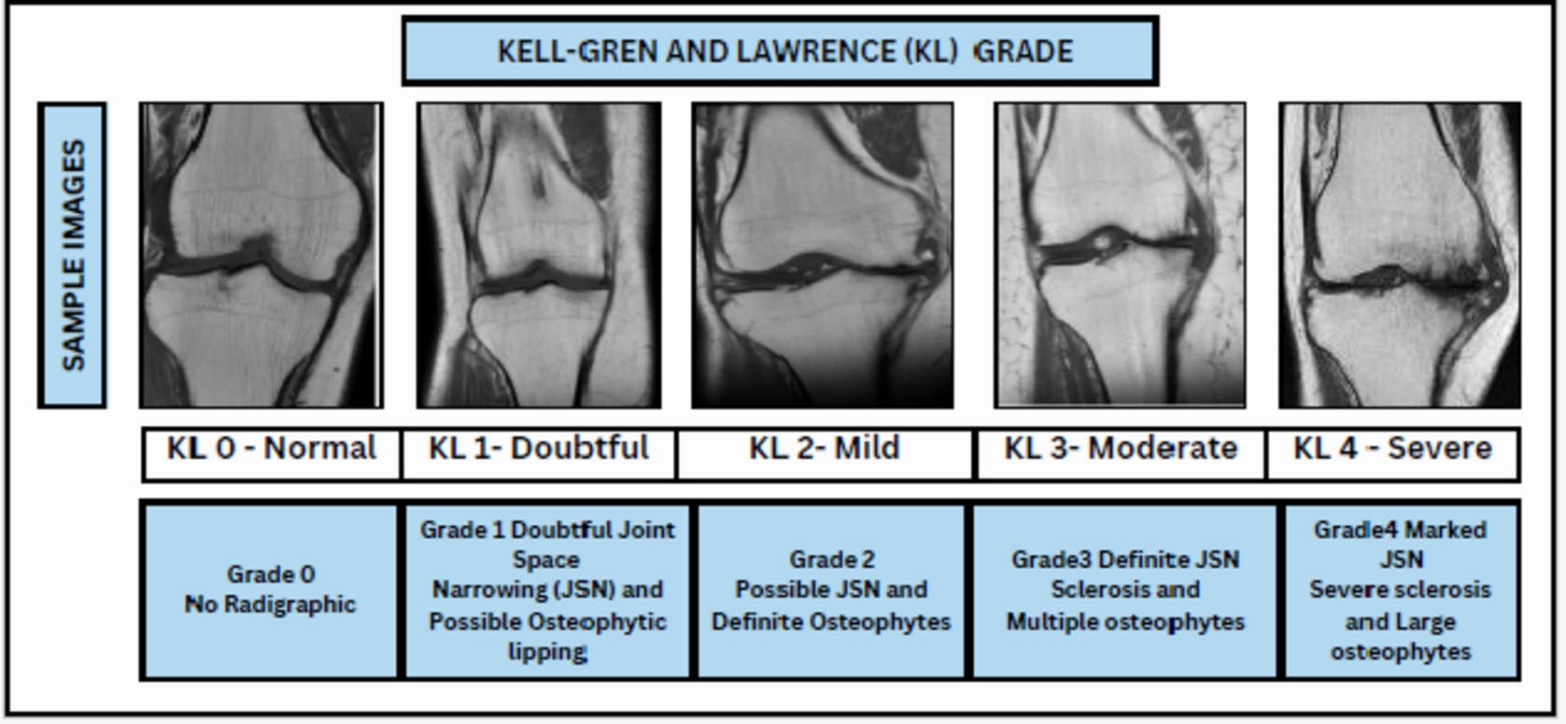
KL- grades distribution of Right Knee MRI.

KOA is seen higher in women because of changes in estrogen hormone and knee structure. Anatomical distinctions between males and females that may be pertinent encompass variations in tibial condylar size, femur width, patellar thickness, and quadriceps angles^4^. Simple radiography (X-rays) is commonly used to assess KOA. Radiologists use a 5-point scale Kell-gren and Lawrence (KL) Scale ranging from 0 pointing to a normal condition to 4 pointing to a severe condition to grade the severity based on x-ray scans. As per KL grading, the radiographic manifestations of osteoarthritis^5,6^are illustrated in Fig. 2. In order to detect initial indications of KOA, various medical imaging techniques are accessible, such as X-ray Scans^7^MRI^8^, CT scans^9^, Ultrasound^10^However, x-ray scans have constraints in capturing alterations in the early stages and issues related to soft tissues. To address these constraints, MRI offers a more thorough assessment of both bone and soft tissues, representing a significant advancement in the diagnosis and understanding of KOA. Moreover, solely relying on X-rays for diagnosing KOA can lead to underestimating the condition and delaying treatment for symptomatic patients. Symptomatic KOA may be present even when X-ray images appear normal. To ensure accurate diagnosis and effective management of the captured images, clinicians should consider additional imaging modalities like MRI and comprehensive evaluation of symptoms, physical examination, and patient history. Three-dimensional MRI images offer a comprehensive view of the entire knee joint, enabling visualization of all tissues. However, the manual detection of cartilage degradation and biomarkers in these images is time-consuming^11^. To address this problem, deep learning techniques are being explored to automate and streamline these processes and ensure more efficient and accurate analysis of 3D MRI images for knee assessment.

The objective of this study is to expedite and improve the diagnostic process for knee osteoarthritis (KOA) through the application of advanced deep learning techniques. Traditional diagnostic methods are time-intensive, often leading to delays in patient care. To address this, we employed pre-trained models and integrated multiple methodologies to develop a faster and more accurate diagnostic approach. Furthermore, this study includes the prediction of grade-wise accuracy, with Grade 4 exhibiting the highest accuracy among all grades. This finding suggests that this approach has significant potential in aiding the prevention of knee replacements by facilitating earlier and more precise diagnosis.

The primary aspects related to predicting KOA in this research study are specified as research objectives 1, 2, and 3.

1. In this research, four pre-trained models, namely CNN, AlexNet, ResNet34, and ResNet50, were utilized. Subsequently, a deep stacking ensemble technique was implemented to improve accuracy.
2. Datasets collected for KOA commonly encounter limitations in terms of insufficient recognition of soft tissue and severity labelling, which hampers the advancement and assessment of effective deep learning algorithms. This dataset seeks to address this challenge by offering a comprehensive and diverse collection of MRI images, encompassing individuals across a spectrum of OA severity levels.
3. To enhance the performance, a deep stacking ensemble technique consolidates predictions from the previously mentioned base models.

## Related work

The current investigation into utilizing deep learning for osteoarthritis prediction through knee MRI scans is a modern research topic. Over the past few years, deep learning methodologies have been employed on MRI scans for the identification and characterization of osteoarthritis. This section provides an in-depth assessment of related literature in the area of applying deep learning techniques to diagnose osteoarthritis through the investigation of knee X-ray and MRI images.

Antony, et al^12^. have used VGG-16, BVLC, CaffeNet for predicting KOA severity based on KL grading. A total of 8892 knee joint x-ray scans from the Osteoarthritis Initiative (OAI) dataset were employed in the study, with the distribution across K0, K1, K2, K3, and K4 grades as follows: 3433 X-ray scans for K0, 1589 for K1, 2353 for K2, 1222 for K3, and 495 for K4. The study reported a mean squared error of 0.504.

Chen, et al^13^. employed the YOLO2 Model for the completely automated identification of knee joints. The study utilized a dataset from the OAI, comprising a total of 4130 X-ray scans. They investigated various fine-tuned models for classification purposes, such as VGG, ResNet, and DenseNet. The highest accuracy they achieved in their study was 69.7%.

Leung, et al^14^. designed a ResNet34 model to autonomously predict the severity of KOA using KL grading. In their investigation, they utilized X-ray image data from 728 patients sourced from the OAI. The highest accuracy attained in their study was 87%.

In a different study, Faster R-CNN was employed^15^. A dataset comprising a total of 2770 X-ray scans, obtained from a hospital, was utilized. The accuracy achieved in their analysis was 82.5%.

An additional study focused on object detection and automated classification of KOA^16^, employing a dataset of 4796 patients’ x-ray images obtained from the OAI. The investigation utilized YOLOv5, VGG16, and ResNet for fully automated KOA detection, yielding an accuracy of 69.8%.

Tiulpin, et al^17^. constructed a Deep Siamese Convolutional Neural Network for predicting knee osteoarthritis based on KL grading. They employed a combined total of 18,376 X-ray scans from the Multicenter Osteoarthritis Study (MOST) for training and 2,957 X-ray scans from the Osteoarthritis Initiative for testing. The accuracy achieved in their study was 93%.

Pedoia, et al^18^. suggested a deep learning algorithm for identifying KOA using MRI Scans. They employed the DenseNet network for the detection task, utilizing a dataset comprising 4,384 subjects with T2 sequence MRI scans sourced from the OAI^19^. The deep learning-based approach achieved an accuracy of 83.4%.

Another study based on automatic detection of KOA severity based on KL Scheme^20^. Faster R-CNN and VGG-16 were employed for the detection of severity, utilizing Posterior-Anterior (PA) and Lateral (LAT) MRI scans. The dataset, sourced from the MOST, included 9,739 scans from 2,802 patients. Out of these, 2,040 MRI scans were allocated for training purposes, 259 for validation purposes, and 503 for testing purposes. The achieved accuracy for the Posterior Anterior (PA) and Lateral (LAT) MRI scans was 71.9%.

Thomas et al^21^. used a convolutional Neural Network model from assessing knee OA severity through x-ray images. The training dataset contained 32,116 images, with 4,074 images utilized for tuning and 4,090 for testing. The reported accuracy of the model was 71%.

In conclusion, numerous studies have investigated the application related to deep learning algorithms in the diagnosis of osteoarthritis through knee X-ray and MRI images. However, our review of the existing literature exposes a notable research gap. Earlier studies focused on the identification of osteoarthritis using knee x-ray images have indicated suboptimal accuracy levels. In order to achieve high-performance outcomes, advanced methodologies are necessary for the identification of osteoarthritis.

## Proposed methodology

**Fig. 3:**
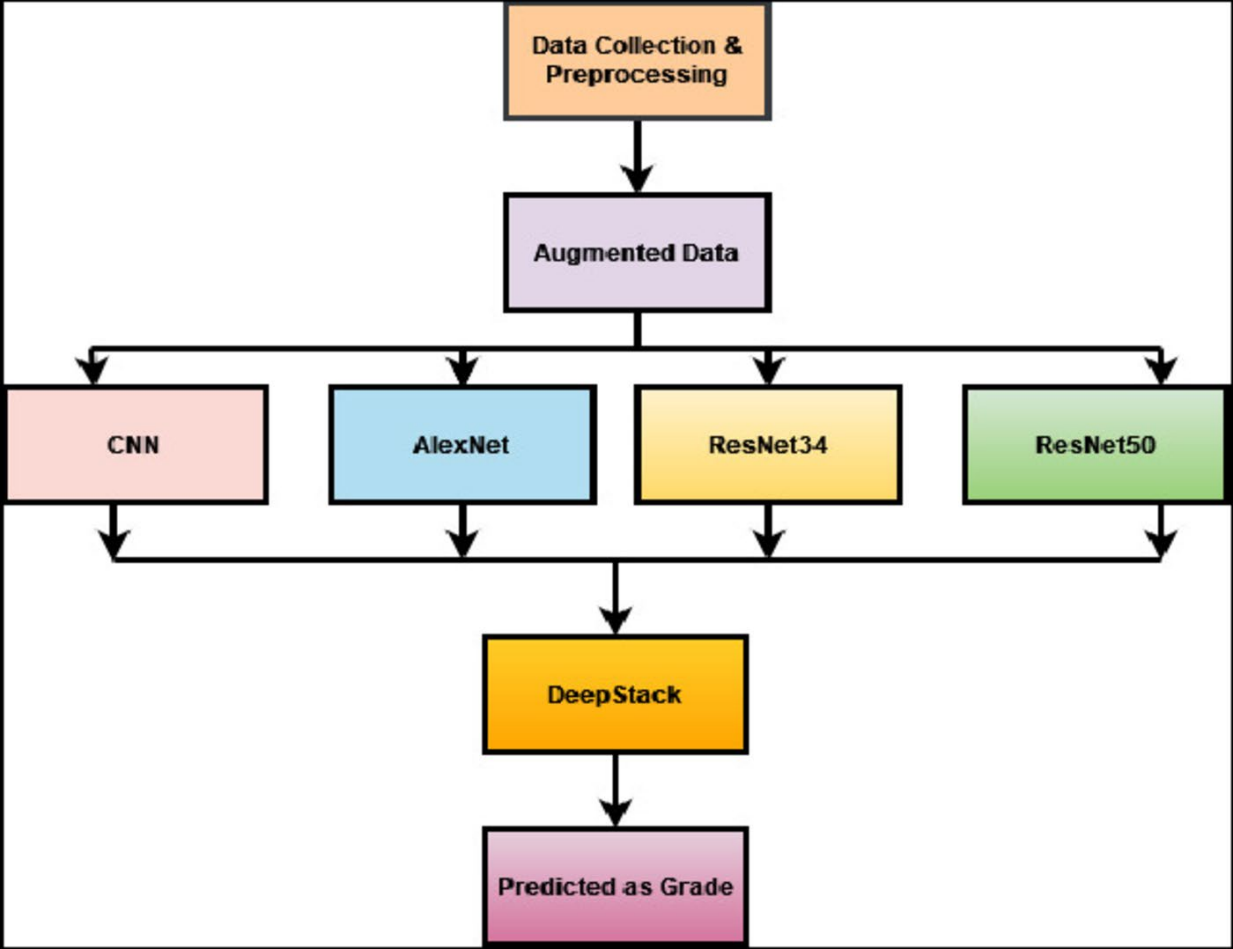
KL- grades distribution of Right Knee MRI.

This section introduces a detailed methodology to accomplish the objectives of the study. The structure of the proposed Deep-Stack model for identifying knee osteoarthritis in buildings is illustrated in Fig. 3. We confirm that all research was performed in accordance with relevant guidelines/regulation, research participants performed in accordance with the Declaration of Helsinki.

### Data collection & preprocessing

The taken Dataset for this particular research was collected from two diagnostic centers which are Dr. Navneet Imaging & Path Lab and Kamal diagnostic center and scrutinized by an experienced doctor affiliated with Mahatma Gandhi Hospital, Jaipur. In the dataset obtained, MRI scans of 1530 individuals are available in DICOM format. Everyone’s MRI comprises approximately 130 to 140 slices, depicting views of the knee from various angles. For this study, the T1 core view, which provides a frontal perspective of the knee, was chosen for analysis. Usually, the early signs of osteoarthritis show up at the age of 45 or above, henceforth, the dataset ensures the presence of persons of the expected age i.e. 45 and above. After performing an age filtering, it was observed that a total of 720 persons were found of age 45 and above. A team of certified and experienced doctors and radiologists gave physical observations for MRI scans of 720 persons. According to their observations, a total of 198 persons having osteoarthritis were segregated into 5 grades according to KL scheme. The collected MRI scans of the diagnosed osteoarthritis patients include both left and right knee scans which is described in Table 1. Therefore, a flip operation was performed on left knee scans to align with the right knee scans^22^.

**Table 1:** Total number of patients and images.

GRADE	NUMBER OF PATIENTS IN LEFT LEG	NUMBER OF PATIENTS IN RIGHT LEG	TOTAL	NUMBER OF IMAGES
KL-0	31	29	60	250
KL-1	22	19	41	200
KL-2	32	29	61	150
KL-3	21	8	29	100
KL-4	5	2	7	50

There are multiple views present in MRI scans of one individual, but the focus remains on T1-cor view.

**Fig. 4:**
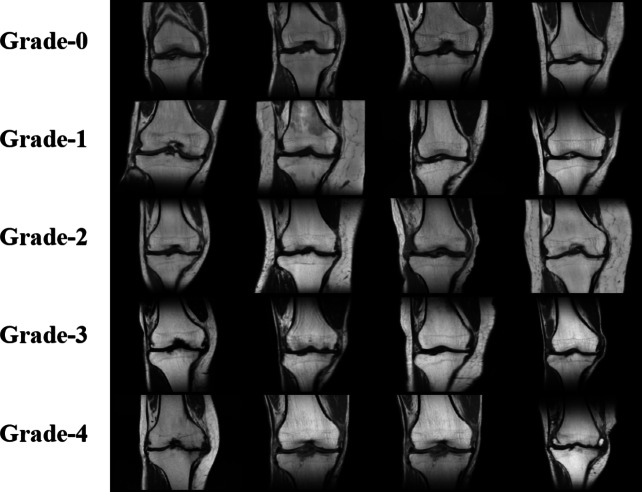
Sample MR Images for Each Grade

**Fig. 5:**
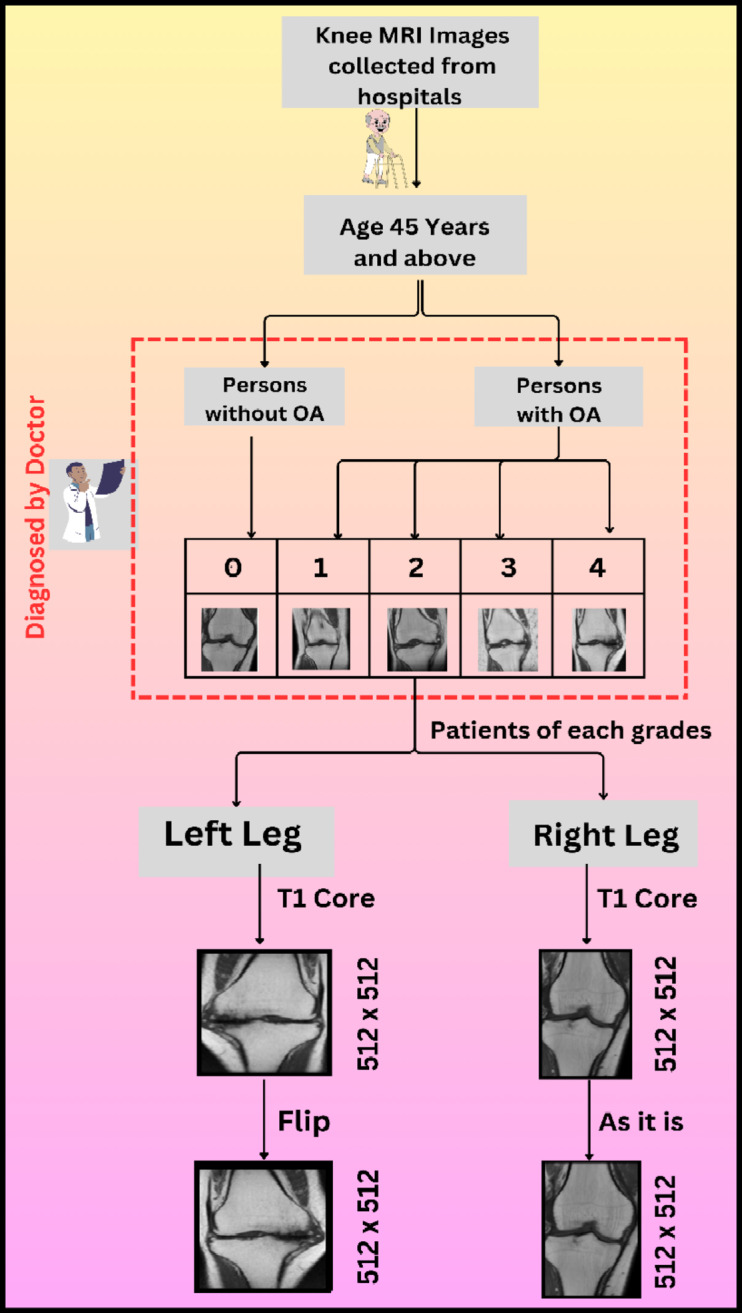
Process diagram of data preprocessing.

A set of 3–4 clear scans of everyone were selected and saved in 512 × 512 JEPG format with the help of Micro DICOM Viewer software. Sample MRI images of each grade are shown in Fig-4. The Final dataset comprises of 750 knee MRI scans. After that, data augmentation was performed for effective training of the model which results in a better performance and removal of certain restrictions like lesser number than desired images. A series of several data augmentation operations were applied to generate 10000 augmented knee MRI scans. Data preprocessing presented in Fig. 5.

### Data augmentation

The process of gathering and preparing extensive data sets for training purposes can incur significant costs and consume substantial time. Employing data augmentation methods enhances the efficiency of smaller data sets, significantly diminishing the reliance on extensive data sets within training setups. Deep learning models heavily lean on diverse and voluminous data sets to foster precise predictions across different scenarios. This augmentation also serves to mitigate overfitting issues. Data augmentation supplements the generation of varied data instances, thereby aiding in refining a model’s predictive accuracy. In this investigation, data augmentation methods were utilized, resulting in the generation of 2000 augmented images for each grade.

### Deep stacking ensemble technique (Deep-Stack)

**Fig. 6:**
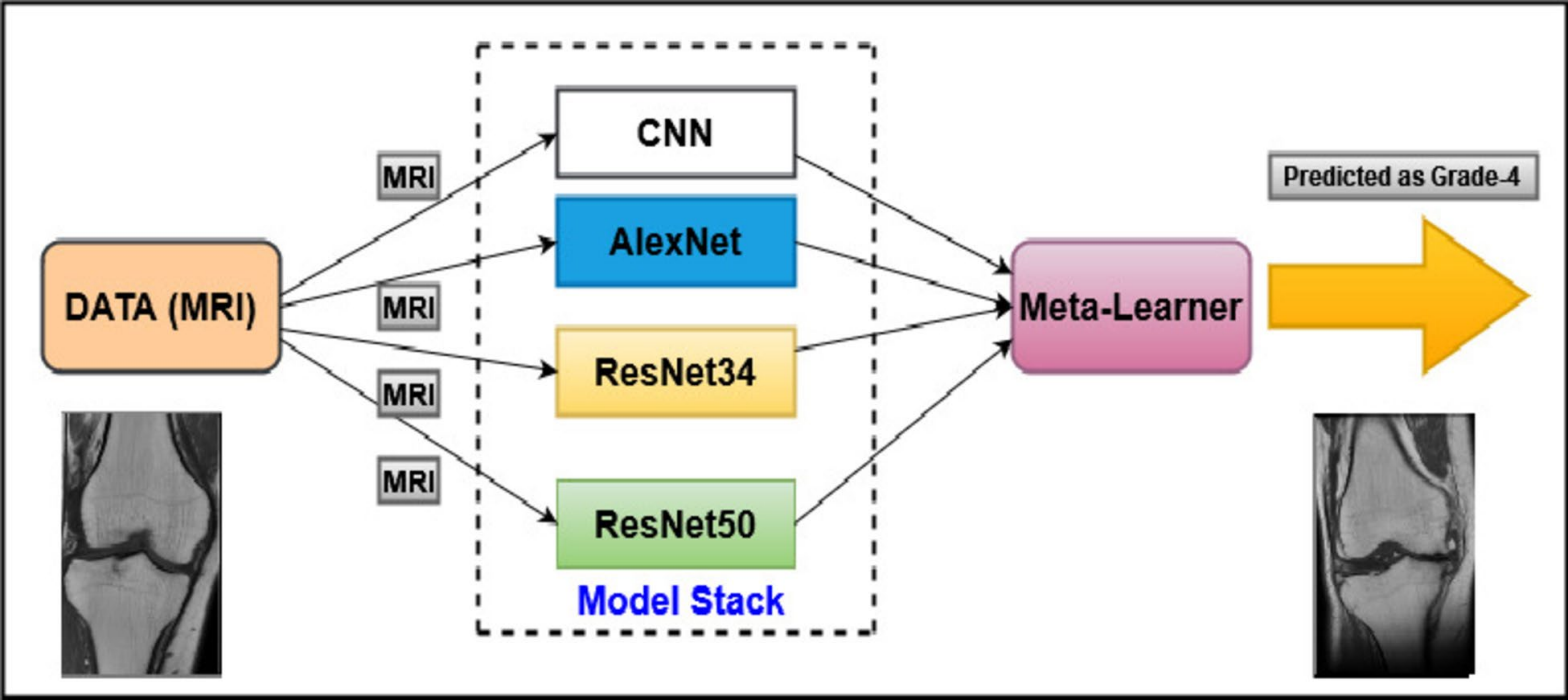
Architecture of deep stacking Model.

Deep-Stack involves utilizing the outputs of base-learners to educate a meta-learner a model that combines previously learnt models, thus enabling it to learn the most effective method for combining the predictions made by the base-learners^23^. The presented research is based on four models, specifically CNN, AlexNet, ResNet34, and ResNet50, all trained on the same dataset but possibly employing different architectures or initialization parameters. These models are subsequently integrated into a Dirichlet Weighted Average Ensemble using the DeepStack library. Following integration, weights are assigned to each model based on its performance metrics, often employing techniques such as the Dirichlet Weighted Average Ensemble approach. This method computes weights that reflect the comparative effectiveness of the models, giving more weight to those demonstrating higher accuracy. After determining these weights, predictions from each model are combined using them to generate the final ensemble prediction. This weighted combination guarantees that models with superior predictive accuracy contribute more significantly to the ultimate outcome. Ultimately, Meta-learning involves developing algorithms that enable AI systems to learn how to learn. These systems are designed to adapt to new tasks and enhance their performance over time without requiring extensive retraining and the process of assigning weights to the ensemble aims to maximize predictive performance, and Overall process described in Fig. 6.

In the proposed study, the Deep-Stack Ensemble consists of four distinct models, all these models are base learners, and all these models are implemented utilizing the Keras Sequential API, as detailed in sub-Sect. 3.3.1, 3.3.2, and 3.3.3 and architecture of models described in Table 2.

#### CNN

The Convolutional Neural Network (CNN) represents an advanced iteration of artificial neural networks (ANN) predominantly employed for feature extraction from matrix datasets with grid-like structures^24^. In the CNN model, four layers have been incorporated, featuring progressively increasing filters (16, 32, 64, 128) with a kernel size of (3, 3) and Rectified Linear Unit (ReLU) as the Activation Function. Additionally, Max Pooling layers with a pool size of (2, 2) are employed. The sequential increase in filters serves to facilitate hierarchical feature extraction, enabling the network to capture spatial hierarchies and progressively complex patterns. To address the overfitting issue, a Dropout Layer with a rate of 0.25 has been integrated.

#### AlexNet

AlexNet is a Deep Neural Network architecture designed for image processing, introduced in 2012. Krizhevsky et al^25^. projected AlexNet model that enhanced the learning capabilities of Convolutional Neural Networks by increasing their depth and employing multiple procedures for parameter optimization^26^. The AlexNet architecture is utilized with a modified stride parameter, as it plays a role in defining the filter size while traversing the input. The use of a stride value of 4 results in a reduction of spatial dimensions in successive layers, potentially leading to a more concise representation of features. This adjustment in the AlexNet implementation has implications for the receptive field and the size of feature maps, impacting the network’s capacity to capture various layers of abstraction from the input image dataset.

#### ResNet34 & ResNet50

In current study, the ResNet34, derived from the ResNet^27^architecture, is implemented with pre-trained ImageNet weights. The model undergoes fine-tuning using the Adam optimizer with a learning rate set to 0.0001. To mitigate the risk of overfitting, a decay strategy is implemented through Cosine Decay, with decay steps configured at 10,000. While ResNet50 and ResNet34 share a similar architectural foundation^28^, their differing depths and complexities allow them to extract different levels of features from the input data. ResNet50, with its deeper architecture, can capture more intricate patterns, while ResNet34 might excel at capturing fundamental features. By combining these models, we aim to achieve a more comprehensive representation of the data. Ensemble multiple models, even with similar architectures, has been shown to improve generalization and reduce overfitting. The combination of ResNet50 and ResNet34 can help mitigate the impact of noise or biases present in individual models. Our experiments have demonstrated that the ensemble model consisting of ResNet50 and ResNet34 consistently outperforms individual models on our validation set.

**Table 2:** Parameters of four base models.

CNN	AlexNet	ResNet34	ResNet50
Input Data (64, 64, 3)	Input Data (64, 64, 3)	Input Data (64, 64, 3)	Input Data (64, 64, 3)
Conv2D_1 (16 filters, 3 × 3, ReLU)	Conv2D: 96 filters, kernel size (11, 11), strides of 4 MaxPool (2,2), strides of 2	ResNet34 (pre-trained)	ResNet50 (pre-trained)
Conv2D_2 (32 filters, 3 × 3, ReLU) MaxPool (2,2)	Conv2D: 256 filters, kernel size (3, 3) MaxPool (2,2), strides of 2	GlobalAveragePooling2D	GlobalAveragePooling2D
Conv2D_2 (64 filters, 3 × 3, ReLU) MaxPool (2,2)	Conv2D: 384 filters, kernel size (3, 3) MaxPool (3,3), strides of 3	Dropout (50%)	Dense (256, ReLU)
Conv2D_2 (128 filters, 3 × 3, ReLU) MaxPool (2,2)	Dense (4096 units, ReLU)	Dense (256, ReLU)	Dense (5, Softmax)
Dropout (25%)	Dropout (50%)	Dropout (50%)	Output (5 classes)
Flatten	Dense (4096 units, ReLU)	Dense (5, Softmax)	
Fully Connected Layer (64 units, ReLU)	Dropout (50%)	Output (5 classes)	
Dropout (25%)	Dense (5 units, Softmax)		
Fully Connected Layer (5 units, Sigmoid)	Output (5 classes)		
Output (5 classes)			

### Experimental setup and model training

The experimental setup, detailed architecture of the utilized models, and the processes for training and testing are elucidated in sub-Sect. 3.4.1, and 3.4.2.

#### Experimental setup

The experimental configuration for predicting the risk of K-OA involved a computer system with distinct specifications. The system operated on a 64-bit Linux OS (Ubuntu 22.04.2 LTS) and was equipped with an Intel i9-10850 K CPU and 64 GiB RAM. The graphics were handled by an NVIDIA GeForce RTX 3080-Ti.

#### Model training and testing

The dataset for the current study has been divided into training, testing, and validation groups in a ratio of 7:1.5:1.5. A total of 150 epochs have been designated for training four models. All four models served as base learners for the meta-learner. The meta-learner constituted a fully connected neural network layer responsible for consolidating the predictions from each sub model and undergoing supplementary training to attain the ultimate outcome.

### Performance metrics

To measure the performance of the implemented models, a confusion matrix has been employed, considering the following classes for evaluation: The classification categories used for sample prediction can be summarized as follows: A true positive (TP) denotes a positive scan, with the model making an accurate positive prediction. A false positive (FP) denotes a negative scan, yet the model erroneously predicts it as positive. A true negative (TN) indicates that a scan is negative, and the model correctly predicts it as negative, while a false negative (FN) indicates that a scan is positive, but the model erroneously predicts it as negative.

In accordance with the summary, accuracy is computed using the expression^29^ provided in Eq. 1. The efficiency of the model is assessed using accuracy.

Accuracy = (TP + TN) **/** (TP + FP + TN + FN) (1).

Precision evaluates the proportion of events predicted as positive by the model that are truly positive. It is calculated by dividing the number of true positive predictions by the sum of true positives and false positives^30^. The computation of Precision follows the expression outlined in Eq. 2.

Precision = TP **/** (TP + FP) (2).

Recall, also known as sensitivity, represents the ratio of correctly identified positive images to all positive cases. The computation entails dividing the number of true positive predictions by the combined sum of true positives and false positives^31^. The determination of Recall follows the formula outlined in Eq. 3.

Recall = TP **/** (TP + FN) (3).

The F1 score serves as a comprehensive metric for evaluating the overall accuracy of a model. A higher F1 score signifies greater efficiency in the model^32^. The calculation of the F1 score is carried out using the formula outlined in Eq. 4.

F1 score = 2 x (Precision x Recall) **/** (Precision + Recall) (4).

## Result analysis

The entire analysis is partitioned into two phases: initially, the training and testing of base learners to assess accuracy individually with each base learner, and secondly, the generation of meta-learners using the deep ensemble technique for evaluating accuracy.

The performance of the four base learners is illustrated in Table 3, providing a comparative assessment using the respective metrics. The CNN model demonstrates a test accuracy of 84.79%, accompanied by precision, recall, and f1 score values of 86%. The AlexNet model surpassed the CNN model in terms of test accuracy, achieving a score of 85.66%. Additionally, the ResNet34 model exhibited a higher test accuracy than the AlexNet model, registering at 95.39%. The most favorable outcomes were obtained with the ResNet50 model, achieving an accuracy of 95.73, along with precision, recall, and f1 score values at 96%. The comparative accuracy based on different grades has been depicted in Table 4 for all four models, and it was observed that the ResNet50 model attained the highest accuracy across all grades, reaching 94.33% for Grade 0, 91% for Grade 1, 94.66% for Grade 2, 98.33% for Grade 3, and 100% for Grade 4.

**Table 3:** Performance of models.

Metrics	CNN	AlexNet	ResNet34	Resnet50
Overall test accuracy	84.79	85.66	95.39	95.73
Precision	86.00	86.00	93.00	96.00
Recall	86.00	86.00	93.00	96.00
F1-Score	86.00	86.00	93.00	96.00

**Table 4:** Grade-wise accuracy of models.

Grade	CNN	AlexNet	ResNet34	Resnet50
0	74.33	79.66	87.00	94.33
1	80.66	77.33	86.33	91.00
2	82.33	81.33	94.66	94.66
3	93.00	91.66	96.66	98.33
4	97.66	98.33	100.00	100.00

**Fig. 7:**
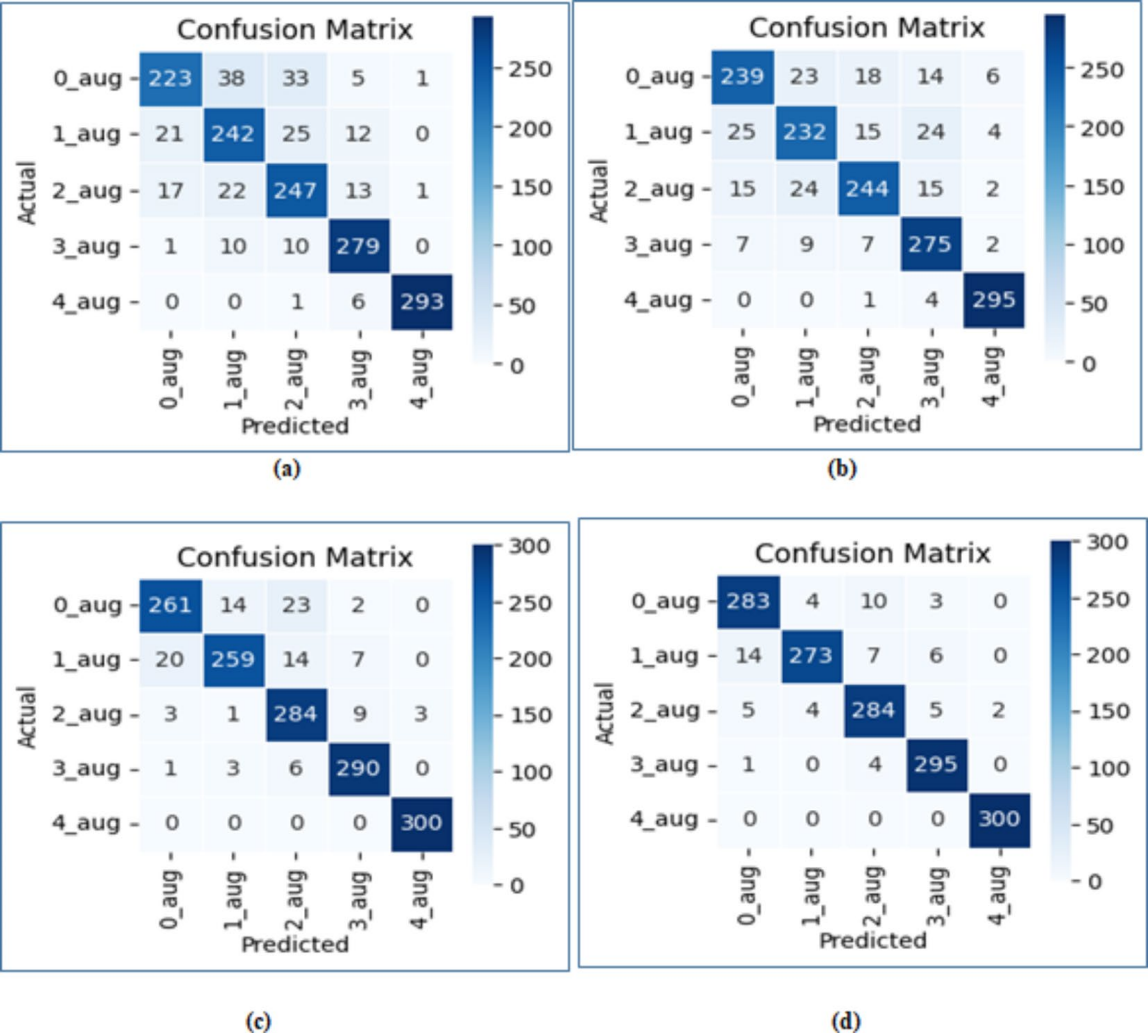
Confusion matrix of (**a**) CNN (**b**) AlexNet (**c**) ResNet34 (**d**) ResNet50.

The acquired results can be corroborated in a similar manner through the examination of the confusion matrix. The confusion matrix for the CNN model is illustrated in Fig. 7(a), where out of 300 samples, the CNN model accurately classified 223 samples for grade 0, while misclassifying the other grades. Figure 7(b) shows the confusion matrix for the AlexNet model. Out of 300 samples, the model correctly classified 239 samples as grade 0, but misclassified the other grades. The ResNet models achieved correct classifications for all samples in grade 4. It is evident from Fig. 7(c) and 7(d) that ResNet34 and ResNet50 models accurately classified the maximum number of samples for each grade.

**Fig. 8:**
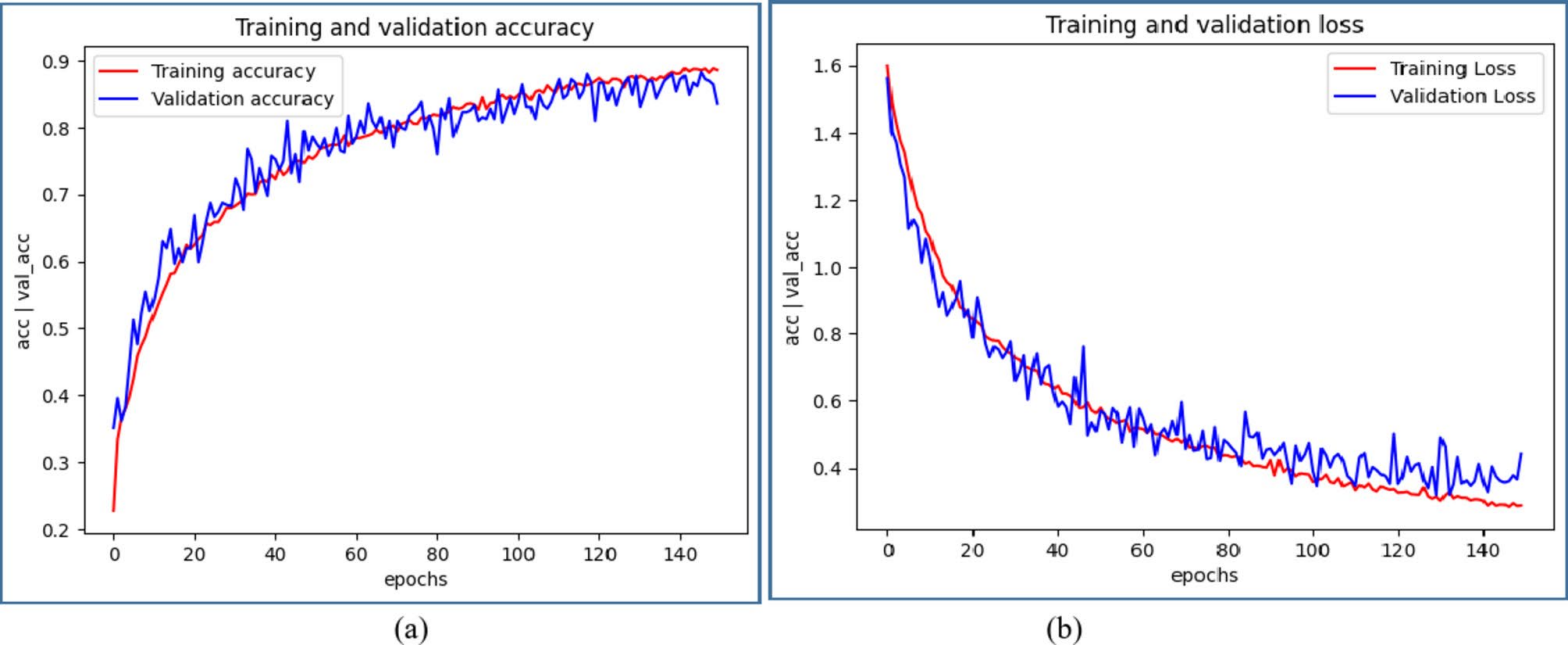
Comparative Analysis of training and validation (**a**) accuracy (**b**) loss for CNN Model

**Fig. 9:**
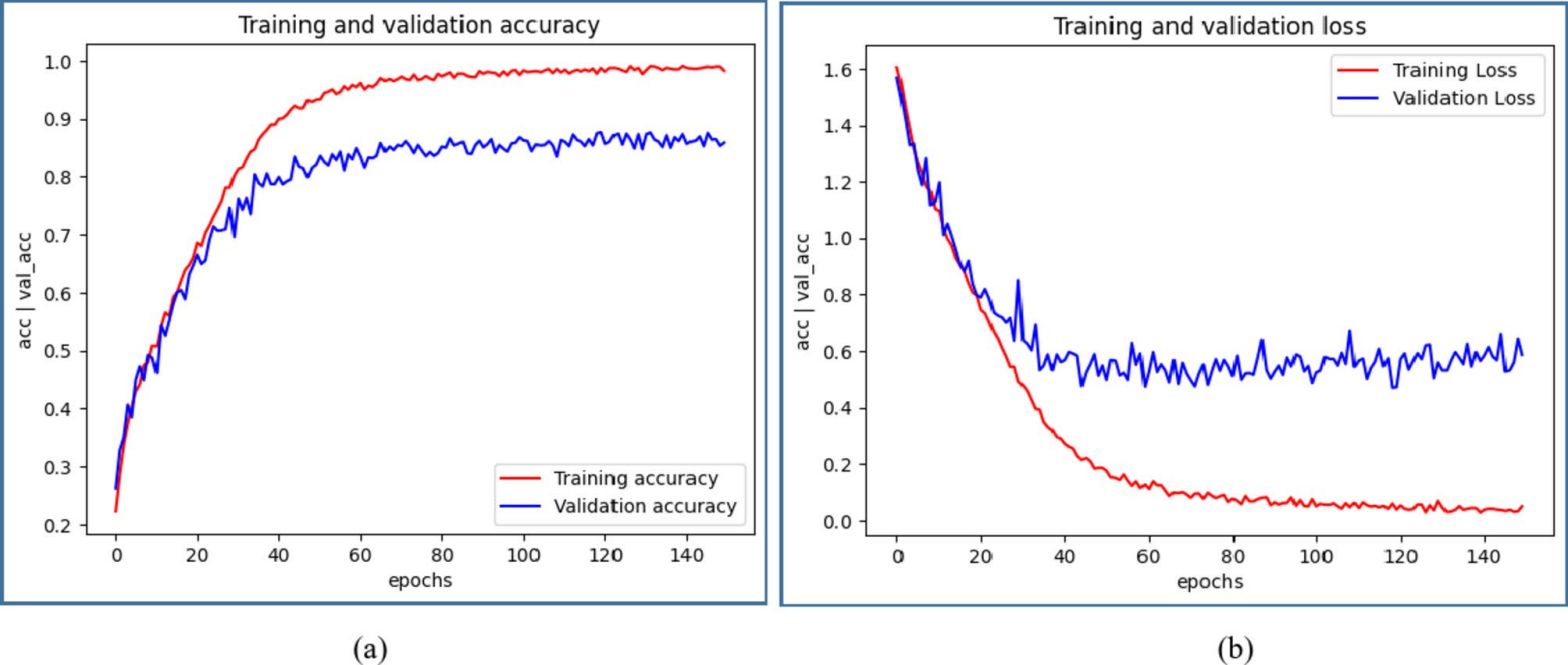
Comparative Analysis of training and validation (**a**) accuracy (**b**) loss for AlexNet Model.

**Fig. 10:**
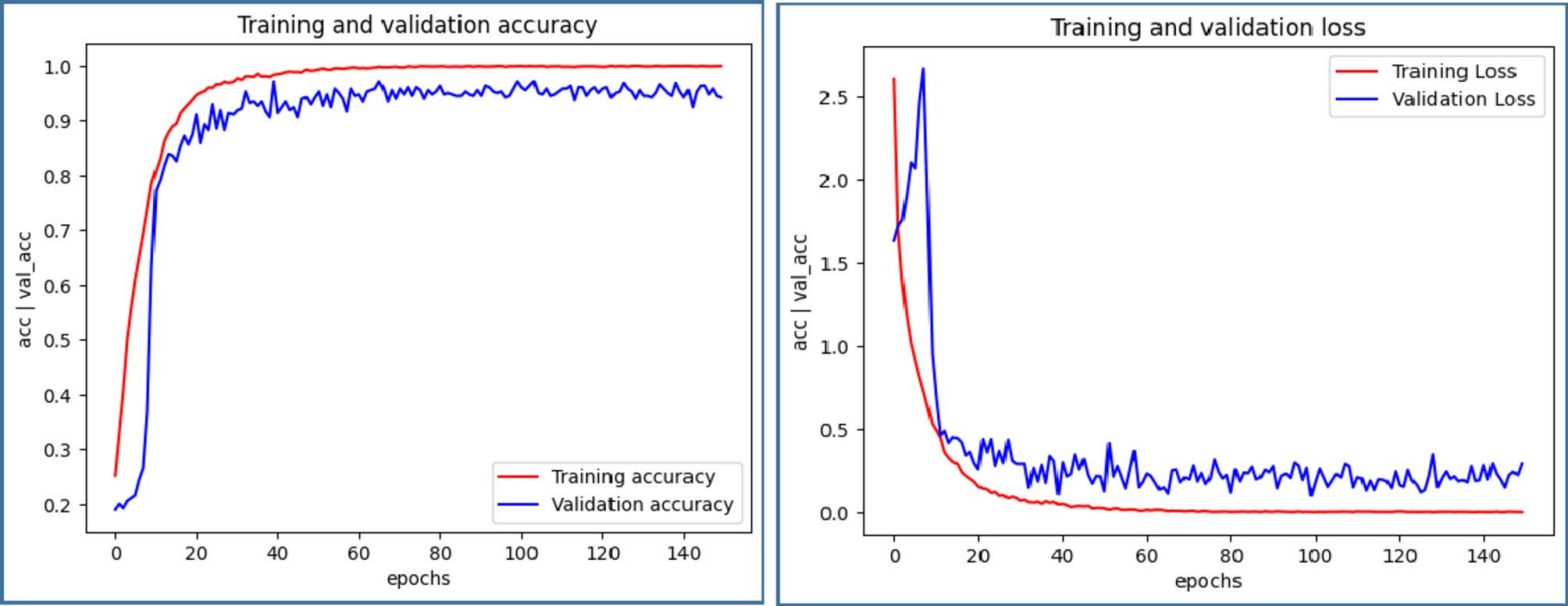
Comparative Analysis of training and validation (**a**) accuracy (**b**) loss for ResNet34 Model.

**Fig. 11:**
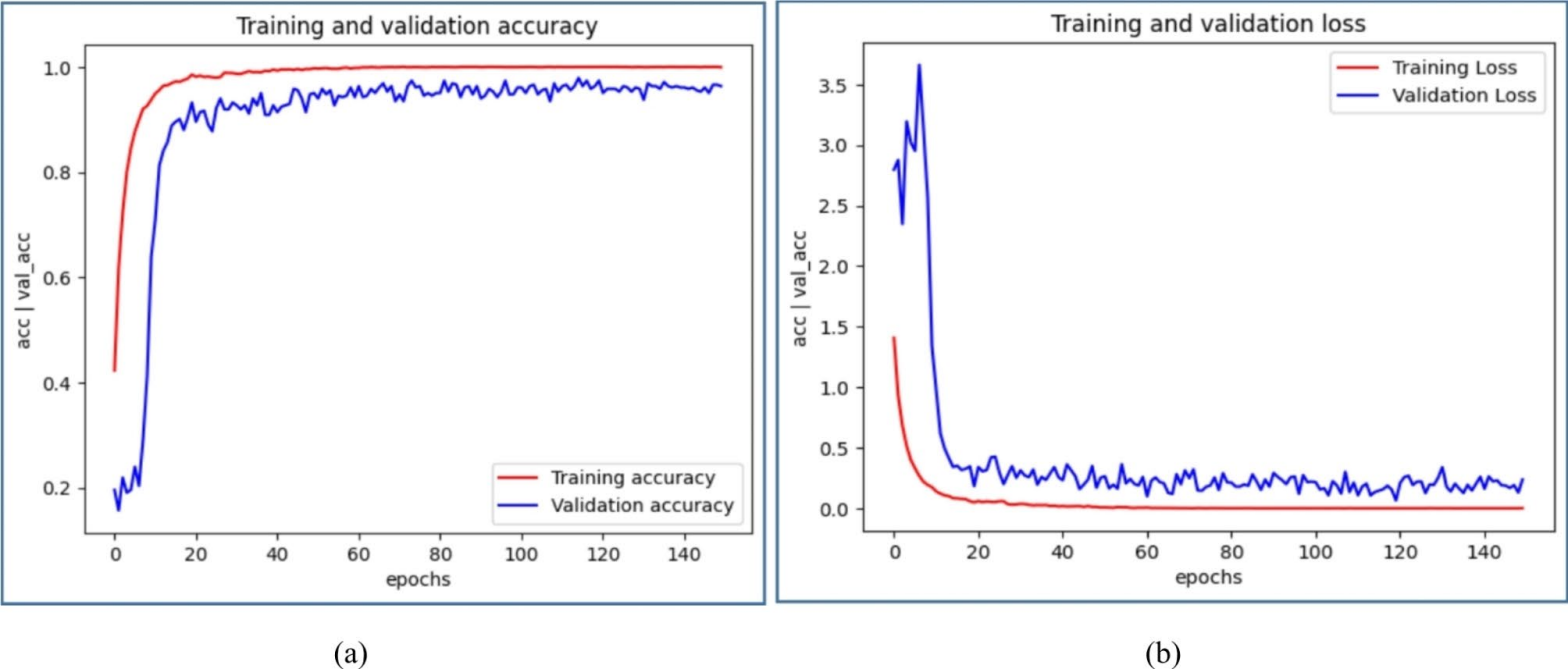
Comparative Analysis of training and validation (**a**) accuracy (**b**) loss for ResNet50 Model.

Figures 8 ,9, 10, 11 illustrate graphical representations of training, validation accuracy, and validation loss for all deep learning models, facilitating a comparative analysis. Training and validation accuracy plots are presented in Figures 8(a), 9(a), 10(a), and 11(a). These plots clearly indicate that as the number of epochs increases, both training and validation accuracy improve. Similarly, Figures 8(b), 9(b), 10(b), and 11(b) present the plots of training and validation loss, demonstrating a reduction in training and validation loss values with an increase in epochs. The conducted work suggests that with each epoch, the neural network becomes more proficient by learning from patterns in the provided MRI scans. This continuous learning contributes to optimal performance through adjustments in the network weights after each learning iteration.

A deep stacking ensemble using a Dirichlet Distribution is implemented, incorporating the generation of meta-learners through the deep ensemble technique for evaluating accuracy. In this context, four models were used: CNN, AlexNet, ResNet34, and ResNet50, with individual accuracies of 84.79%, 85.66%, 95.39%, and 95.73%, respectively. These four separate models, identified as base learners, are included in the ensemble, each assigned specific weights and accompanied by accuracy scores. The Dirichlet ensemble employs a Dirichlet distribution to compute weights, leveraging the probabilistic characteristics of the distribution to dynamically assign weights according to the performance of each individual model. The models were assigned weights of 0.0013, 0.0212, 0.4373, and 0.5402, determined by their perceived contributions to the ensemble, shown in Table 5. Subsequently, the predictions of each model were multiplied by their respective weights, and the outcomes were consolidated to generate a unified prediction for each data instance. Normalization was applied to ensure the predictions were confined within the [0, 1] range. The accuracy reported for the final deep stacking model was 99.71%. Therefore, the deep stacking ensemble yields highly accurate predictions for MRI images.

**Table 5:** Automatic assignment of weights and accuracy.

Model	CNN	AlexNet	ResNet34	ResNet50
Weights	0.0013	0.0212	0.4373	0.5402
Accuracy	0.9280	0.9774	0.9955	0.9961

In this study, we conducted an ablation analysis to assess the impact of different model combinations on the ensemble accuracy. Initially, we applied an ensemble model incorporating all four base models, which yielded an accuracy of 99.71%. To further investigate the contribution of each model, we created several ensembles by excluding one model at a time.

First, we evaluated an ensemble of three models: AlexNet, ResNet34, and ResNet50, achieving an accuracy of 99.19%. Next, we formed another ensemble with CNN, ResNet34, and ResNet50, resulting in an accuracy of 98.07%. Following this, we tested an ensemble of CNN, AlexNet, and ResNet50, which produced an accuracy of 97.82%. Lastly, we examined an ensemble consisting of CNN, AlexNet, and ResNet34, which achieved an accuracy of 97.54%. The results for all model combinations are presented in Table 6.

**Table 6:** Automatic assignment of weights and accuracy subset of the four networks.

Base-Model	Weight	Accuracy-Score	DirichletEnsemble Accuracy
AlexNet	0.239	0.9792	0.9919
ResNet-34	0.1598	0.9931	
ResNet-50	0.6012	0.9967	
CNN	0.224	0.9281	0.9807
ResNet-34	0.1746	0.9931	
ResNet-50	0.6014	0.9967	
CNN	0.228	0.9281	0.9782
AlexNet	0.1618	0.9792	
ResNet-50	0.6102	0.9967	
CNN	0.238	0.9281	0.9754
AlexNet	0.1629	0.9792	
ResNet-34	0.5991	0.9931	

Our findings indicate that the highest ensemble accuracy is attained when all four base models are included. From the perspective of knee osteoarthritis prediction, achieving the highest possible accuracy is critical for effectively preventing knee replacement surgeries. Therefore, utilizing all four models in the ensemble is essential for optimal performance.

## Comparisons to current state of the Art Research

Table 7 presents a comparative analysis of the performance of our proposed study in comparison to other leading studies. Studies published between 2021 and 2024 have been chosen to ensure a comprehensive and balanced comparison.

**Table 7:** The performance evaluation of our proposed study in detecting osteoarthritis through knee X-ray images, compared with other state-of-the-art studies.

Ref. No.	Year	Classification Techniques	Dataset	Performance Accuracy
Chen, Pingjun, et al. [13]	2019	YOLO2, VGG, ResNet, and DenseNet	4130 X-ray scans (OAI)	69.7%
Leung, Kevin, et al. [14]	2020	ResNet34	728 patient’s x-rays (OAI)	87%
Liu, et al. [15]	2020	Faster RCNN	2770 X-ray scans	82.5%
Dalia, Yuvraj, et al. [16]	2021	YOLOv5, VGG16, and ResNet	4796 patients’ x-ray Scans	69.8%
Tiulpin, Aleksei, et al. [17]	2018	Deep Siamese Convolutional Neural Network	18,376 X-ray scans (Training), 2,957 X-ray scans (Testing) (OAI & MOST)	93%
Pedoia, Valentina, et al. [18]	2019	DenseNet network	4,384 subjects with T2 sequence MRI scans (OAI)	83.4%
Swiecicki, Albert, et al. [20]	2021	Faster R-CNN and VGG-16	9,739 MRI scans (MOST)	71.9%
Thomas, et al. [21]	2020	CNN	40,280 x-ray scans (OAI)	71%
Y. Wang, et al. [33]	2021	CNN + YOLO	4506 x-ray scans (OAI)	95%
Yuniarno, et al. [34]	2022	Deep CNN	390 x-ray scans	83%
K. Üreten et al. [35]	2022	Pre-trained VGG-16	710 x-ray scans	90%
B. C. Dharmani et al. [36]	2023	EfficientNet-B1	9739 x-ray scans	89%
J. H. Cueva, et al. [37]	2023	Fine Tuned ResNet-34	4796 x-ray scans (OAI)	61%
Mohammed Abdul et al. [38]	2023	ResNet-101	9786 x-ray scans (OAI)	69%
Patil et al. [39].	2024	Densely connected fully convolutional network	1100 x-ray scans (OAI)	94%
Touahema et al. [40]	2024	Xception Model	5000 x-ray scans (OAI)	95.36%
Jain et al. [41]	2024	High resolution network	x-ray (OAI)	71.74%
**Proposed Method**	**2024**	**Deep Stacking Ensemble with four Base Models**	**10,000 MRI scans**	**99.71%**

## Conclusion

In the current research study, the automated diagnosis of K–OA on the MRI scan dataset was successfully accomplished through the implementation of a highly effective deep stacking ensemble method on base learners. The results indicate a substantial enhancement in performance with utilization of deep stacking ensemble technique on MRI scans as compared to x-ray scans. The implementation of deep stacking ensemble technique on the base learners a higher accuracy result was observed on the dataset MRI scans. In terms of accuracy, the deep stacking ensemble method exhibits superior performance compared to other available methods for automated diagnosis of knee osteoarthritis from MRI scans. The suggested approach creates new possibilities for radiologists and medical practitioners, facilitating a straightforward and early diagnosis of K-OA. This advancement is expected to greatly benefit patients by enabling timely and effective treatment, thereby minimizing the suffering caused by the severity of the disease, which tends to escalate in the absence of timely diagnosis. In the future phase of the project, there will be a continued exploration of additional techniques aimed at achieving higher accuracy, while simultaneously focusing on minimizing complexity and optimizing time efficiency.

## Data Availability

Data cannot be shared openly to protect study participant privacy. If it is required, we can submit dataset as supplementary material. For data access requests related to this study, contact punitapanwar7@gmail.com.
